# 2023 European Kidney Forum: The future of kidney care – investing in green nephrology to meet the European Green Deal targets

**DOI:** 10.1007/s40620-025-02280-y

**Published:** 2025-06-05

**Authors:** Olivier Philippe van Vredendaal, Alicia Bé, Ilaria de Barbieri, Daniel Gallego, Fiona Loud, Giorgina Barbara Piccoli, Fokko Wieringa, Raymond Vanholder

**Affiliations:** 1 European Kidney Health Alliance, Brussels, Belgium; 2 Present Address: Health Practice, DGA Group, Brussels, Belgium; 3 European Dialysis and Transplant Nurses Association/European Renal Care Association, Hergiswil, Switzerland; 4 European Kidney Patients’ Federation, Madrid, Spain; 5 Kidney Care UK, Alton, UK; 6 Nephrology, Centre Hospitalier Le Mans, Le Mans, France; 7 Autonomous Therapies Department, IMEC, Eindhoven, the Netherlands; 8 UMC Utrecht, Utrecht, the Netherlands; 9 Chair WG3, European Kidney Health Alliance, Brussels, Belgium; 10 Nephrology Section, Department of Internal Medicine and Pediatrics, University Hospital, Ghent, Belgium

**Keywords:** Environmental health, Sustainability, Innovation, Transplantation, EU

## Abstract

The European Kidney Health Alliance’s 2023 annual Policy Forum brought together relevant stakeholder groups and examined the impact of the environment on kidney health and of kidney replacement therapies on the environment; the environmental footprint of kidney care; and the role of the European Union (EU) in facilitating the transition to sustainable healthcare. Interventions made by nephrologists, patient representatives, renal nurses, scientists, civil society representatives, medical technology specialists, and EU policymakers and administrators considered themes that included the burden of chronic kidney disease (CKD) in the EU, the link between kidney health and environment, waste production and resource use, organ transplantation, technological innovation, and the role that the EU has to play in addressing the burden of kidney disease and fostering more sustainable healthcare practices. The 2023 European Kidney Forum illustrated the broad consensus among diverse stakeholders operating within the kidney health landscape on the critical need to adopt a more sustainable approach to kidney care in the EU. The Forum also highlighted the cruciality of EU policymakers’ commitment to improving the sustainability of the healthcare sector through EU policies, tools and initiatives. Panel discussions pointed to the strong consensus between different stakeholders in the kidney health domain on the need for action to better prevent chronic kidney disease; improve the EU’s organ donation and transplantation landscape; and recognise and address the substantial environmental impact linked to the management and treatment of CKD.

**Graphical abstract:**

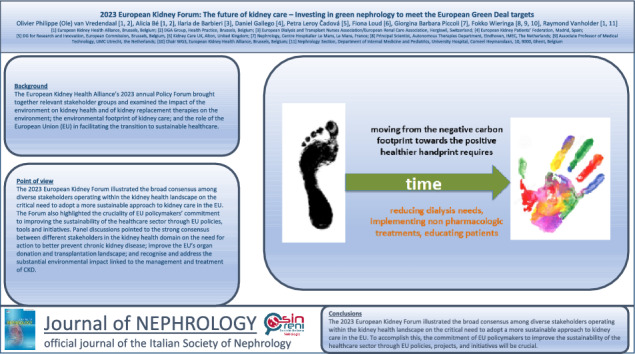

## Introduction

The European Kidney Health Alliance (EKHA) [1] is a non-governmental advocacy organisation defending the case of kidney patients and the nephrological community at the European Union (EU) level. Each year, EKHA organises a Policy Forum bringing together relevant stakeholder groups, including nephrologists, patient representatives, renal nurses, scientists, civil society representatives, medical technology specialists, and EU policymakers and administrators.

The 2023 European Kidney Forum examined the impact of the environment on kidney health and of kidney replacement therapies (KRT) on the environment. The Forum encompassed prospects of reducing the environmental footprint of kidney care—especially dialysis—related to both waste creation and the consumption of resources, and the possible role of the EU in facilitating the transition to sustainable healthcare [2].

Healthcare is among the most polluting economic sectors, accounting for 5.2% of global greenhouse gas (GHG) emissions, substantial water, air and soil pollution, use of fresh water, and waste creation [3, 4], and consuming large amounts of (fossil-fuelled) energy resources [5]. Despite several European countries taking action to reduce healthcare emissions, the healthcare sector was estimated to have contributed 330 megatons of CO2-equivalent emissions in 2020 [6]. Within the healthcare sector, kidney care is a major polluter, as well as resource consumer.

Europe is observing an increase in climate-change-related health hazards, such as heatwaves and extreme weather events. Given existing patterns of rises in global temperatures, such weather events are likely to increase in frequency (and severity) in the future. Nevertheless, ambitious mitigation measures are needed to limit the global temperature rise to less than 1.5 °C above pre-industrial levels and avoid reaching points of no return [4]. Therefore, a paradigm shift towards environmentally considerate kidney care is urgently needed and should be achieved through sustainable technological innovation as well as promoting more environment-friendly, efficient and cost-effective approaches to kidney care [7–10]. As the Forum highlighted, healthcare practitioners, patients, policymakers, health technology providers and manufacturers should be well-aligned on the urgency of implementing green nephrology approaches in the EU.

Europe is the world’s third-largest economy and a major polluter. As such, it represents a crucial player in the global response to climate change. Europe not only has the responsibility but also the opportunity to drive the transition to a climate-resilient and low-carbon society, including the healthcare sector [4].

### The burden of CKD and need for EU-level action

The two Co-Chairs of the Member of the European Parliament (MEP) Group for Kidney Health at the time of the Forum (see acknowledgements) put the spotlight on the need for ambitious EU action to address CKD’s burden. Over 100 million EU citizens suffer from CKD and 300 million are at risk [11], which will inevitably translate into a growing environmental burden if kidney care does not implement climate mitigation measures. Under the framework of the European Green Deal, efforts are underway to make Europe climate-neutral by 2050 and to reduce greenhouse gas emissions by 55% by 2030 [12]. What is more, the EU’s LIFE Program and the Horizon Europe framework provide the funding crucial for undertaking action, including research, on climate and environment [13, 14]. The ambitious European Green Deal targets must be reflected in all areas of the economy, including the healthcare sector. Currently, the environmental impact of the healthcare sector is often forgotten. Especially that of nephrology, a sector that does not get enough recognition at the EU level.

In addition, sustainable innovation in kidney care also holds the promise of answering patients’ calls for better and more appropriate care options, therefore innovation can both protect the environment and ease the burden that CKD places on the individuals affected. The EU should play a critical role in the area of patient-driven therapies, with a specific focus on the only sensible choice ahead of us, which is investing in environment-friendly healthcare including kidney care. Moving forward, it is crucial to ensure less burdensome, more sustainable and more patient-driven therapies, and the EU can be a global leader in this field.

### Setting the scene: the link between kidney health and environment

Similar concerns loom over kidney healthcare. Haemodialysis accounts for a substantial environmental footprint, consuming vast quantities of natural resources and energy, resulting in substantial greenhouse gas emissions [2, 8, 9, 15, 16]. For an average dialysis unit, water consumption rates can exceed one million litres per year and, globally, 169 billion litres of water are used in dialysis annually, which generally are discarded in the sewer system [12, 15, 17]. Finally, dialysis also generates large amounts of plastic waste, stemming largely from the continued widespread application of single-use plastics, which are usually not recycled but mostly incinerated [2].

Translating these abstract figures into tangible facts, one year of haemodialysis across Europe would be equivalent to the energy consumption and GHG emissions of 2.5 million combustion-engine car return trips from Brussels to Istanbul. The energy consumption of a person on haemodialysis is twice as high as the energy usage of the average European. Furthermore, when considering all haemodialysis treatments in Europe during a one-year period, the volume of freshwater used would be sufficient to fill 10,000 Olympic swimming pools, and the volume of plastic waste generated would be enough to fill 18,000 heavy goods vehicles. These staggering statistics underscore the environmental impact of haemodialysis and highlight the urgent need for more sustainable practices in kidney healthcare.

The alternative to haemodialysis (HD), peritoneal dialysis (PD), holds certain advantages, such as reduced water consumption and less energy usage per patient, and, as a home strategy, reduced movement of patients and personnel [18]. However, these benefits are partly counterbalanced by higher plastic consumption and resultant waste, as well as increased transportation needs. For instance, a single PD treatment may require the transport of PD dialysate bags with a total volume of 16 L or more for 48 h of treatment, compared to a single hemodialyzer and tubing set for HD needed for the same time period. However, a comprehensive Australian life cycle analysis of peritoneal dialysis including an assessment of the carbon footprint of production, transport and disposal, showed lower emissions of continuous ambulatory peritoneal dialysis (CAPD) and automated peritoneal dialysis (APD) than the figures usually accepted for conventional HD [19].

Current CKD management not only has an impact on the environment, but environmental conditions, in turn, also have serious effects on the onset and progression of kidney diseases. In particular, climate change, associated heat waves and environmental pollution can disrupt kidney function due to dehydration, kidney stone development, kidney damage due to fine dust, the spread of vector-carried diseases such as malaria or dengue, and various other mechanisms [2, 20–24]; and can affect kidney care in case of extreme weather events (e.g., when dialysis units are flooded) [25].

The most vulnerable populations, including the poor and minorities, are disproportionately affected by the adverse effects of climate change. These communities often reside in areas with limited green space and in buildings that lack adequate protection against heat. Furthermore, they may have lower access to quality healthcare, leaving them less equipped to cope with the effects of climate change [26, 27].

These challenges underscore the critical necessity for an urgent paradigm shift in kidney care. This shift should encompass a heightened focus on screening, prevention, and transplantation. Moreover, research and innovation should be directed towards developing dialysis systems that consume less water and energy while incorporating the circular treatment of polymer material that involves reuse and/or recycling.

### Kidney patients’ perspective on the current dialysis model

The current model for treating advanced CKD, which emphasises dialysis, is inadequate because it does not exploit the prevailing clinical and technological possibilities, including those with a positive environmental impact. While CKD should be prevented as much as possible, transplantation stands out as the preferred KRT. Yet, major obstacles hold back its implementation: particularly, organisational and political barriers in many countries; flaws in organ preservation, surgical procedures, adherence to prescribed therapies, and prevention and treatment of rejection; and side effects of immunosuppressant medications. Hence, many patients currently rely on dialysis for their survival. Nevertheless, the current model of dialysis, while remaining the standard care practice, should be transformed, particularly because of the considerable negative impact on patients’ capacity to work, their possibilities for social interaction, and their overall quality of life, all related to the need to undergo dialysis several times per week in clinical settings. Having to maintain such treatment regimens has a detrimental effect and is not compatible with living a normal life. Against this backdrop, and considering environmental impact, the need to rethink the treatment of advanced CKD in the present and near future is compelling. Innovative initiatives should centre around longevity, home delivery of care, portable treatment devices, regeneration of dialysate, and durable types of vascular access. Particularly, the current acquiescence to the physical pain created by the cannulation for vascular access puncture should be reconsidered to develop more permanent and painless solutions, with lower risks of infection.

Patients cannot be held responsible for the environmental impact of the treatment that they need for their survival. Instead, governments, policymakers, healthcare professionals, and industry are responsible for collaboratively addressing this challenge. However, kidney patients should be involved in innovative initiatives, with their experience guiding these efforts. These should encompass a broad spectrum, including policy decisions, development and support of projects, and investments to design environment-friendly nephrology practices and to improve quality of care and quality of life of individuals with kidney disease.

### The energy crisis and kidney care

Greenhouse gas emissions related to the high energy needs of dialysis are also indirectly linked to the quality of life and personal situations of people with advanced CKD. Specifically, CKD is more frequent among vulnerable people, such as the poor and minorities [26], and this precarious condition is further exacerbated by natural events, wars, or pandemics [28]. Increases in energy prices, like those in 2022, were extremely challenging for the disadvantaged, especially for those on home dialysis, since patients in many countries were not refunded for these extra costs. A way out of this conundrum could be kidney transplantation, or new, innovative KRTs with lower energy needs. Eco-friendly alternatives to dialysis would not only lessen the burden on our environment but also decrease the financial burden on patients. It is the responsibility of all concerned to consider what provisions should be made to ensure financial and power continuity for home dialysis patients, to create a greater awareness of how kidney health can be preserved, and kidney disease prevented, and to generate more opportunities in the area of transplantation.

### Waste production, resource use and sustainable kidney disease management

The implementation of the green nephrology concept in daily kidney care requires combined efforts at all levels. While the commitment of the healthcare team and providers is crucial to making progress possible, political bodies have a great responsibility to facilitate the transition, priming innovative models of care, updating obsolete legal frameworks, and incentivising research.

**Fig. 1:**
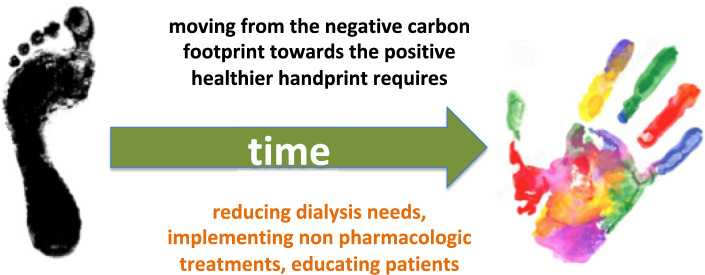
The transformation towards optimisation of resources requires time

Tables 1 and 2 report the main technical and clinical bottlenecks that have been identified as key factors for a more environment-friendly approach to the nephrology field, and which could benefit from political support. While technical issues are relatively easy to address, usually resulting in a favourable economic evolution after an initial investment, addressing the listed clinical issues would require more in-depth reflection on current care-delivery models to be effective. Many interventions aimed at obviating or postponing the need for dialysis are lifestyle measures which also contribute to increasing sustainability (e.g., reduced (red) meat consumption, increased physical exercise) but require trained teams to invest time in information and education, which may not always be feasible given the current trend towards industrialisation of medicine. As a result of this development, physicians can be seen as “therapy deliverers,” who are no longer involved in holistic personalised care via a robust patient-physician relationship. It is thus important that time be allotted for true shared decision making, since decisions concerning treatments should be discussed between patients and physicians at an equal level, and that personalised care programmes be established that offer both short- and long-term advantages for patients and planetary health. However, this also implies that the workflow of nephrologists and overall healthcare teams should be revised. In addition, patients should be informed and empowered to request planet-friendly solutions from their caregivers. Patient and renal nurse organisations could take a lead role in this process. Together with efforts to decrease the need for dialysis, including through non-pharmacologic treatments, such an education process can, in time, help lower the current negative carbon footprint of kidney care, as Fig. 1 illustrates.

**Table 1:** Technological actions, barriers and solutions to improve the environmental impact of dialysis

Issue, references	Main potential actions	Barriers	Potential facilitators at the EU institutional level
Water recycling [52–54]	Reject water from the reverse osmosis system for preparing dialysate is microbiologically safe and can be used for various purposes, with or without desalinization or dilution, and even complies with the definition of drinkable water. Spent dialysate may be used to produce fertilizers	Need for technical implementation of devices to collect wastewater; outdated reverse osmosis systems; lack of funds; lack of awareness	Revising the rules for wastewater reuse. Promoting educational campaigns; establishing funding for implementation
Green energy [52, 55, 56]	Solar or renewable energy can reduce the hospital’s carbon footprint; thermic and kinetic energy of dialysate fluids could be exploited	Need for technical implementation; outdated hospital infrastructure; lack of funds; lack of awareness	Promoting educational campaigns; establishing funding for implementation
Waste management [52, 57, 58]	Dialysis produces a vast amount of plastic waste, partly not in contact with body fluids, and thus recyclable. Recycling may reduce the global burden of plastic. Recycling can allow better use of resources, and reduce contamination and plastic burden	Low availability of recycling facilities; lack of awareness; lack of time for proper waste triage and management. Lack of interest from industry. Legal restrictions on recycling of material that has been in contact with body fluids	Improving the regulations on plastic recycling. Educational campaigns; establishing funding for recycling
Disposable design [59]	The current disposables are not intended to be easily recycled. A better design according to a cradle-to-cradle philosophy is needed for improvement	Lack of collaboration with the industry	Promoting common programs, defining standards of recyclability
Hardware design [59]	The current hardware (dialysis machines and water treatment systems) is not intended to be recycled. A better design according to cradle-to-cradle principles is needed. The lifespan of the dialysis machines needs to be prolonged	Lack of collaboration with the industry. Dialysis machines are not designed for long-term use. Lack of registries specifying the age of dialysis machines	Promoting common programs, defining standards of recyclability. Organising registration of devices
User-friendly and flexible dialysis machines	The current dialysis hardware is bulky and does not allow flexibility to travel or to perform dialysis at work. Current dialysis machines apply single-pass dialysate handling	Lack of interest of manufacturers; lack of investment, lack of research	Rethinking the current dialysis model towards user-friendly and compact systems, recycling dialysis water to decrease water and energy consumption

**Table 2:** Clinical actions, barriers and solutions to improve the environmental impact of nephrology and dialysis

Issue, references	Main potential actions	Barriers	Potential facilitators at the EU institutional level
Nutritional management [58, 60]	Healthy diet is essential for prevention and treatment of all chronic diseases. A well-planned and adapted diet for people with kidney disease has the potential to increase access to kidney transplantation, stabilize kidney function, improve survival	Healthy food is more expensive than ultra-processed food. Shortage of dedicated dieticians while the number of patients with CKD is growing. Lack of specialised dieticians in nephrology	Financial incentives to facilitate the functioning of units for kidney care with a dietician. Establishing educational and follow-up programs. Appropriate diet for people with kidney disease
Promoting exercise [61]	Regular exercise is essential for prevention and treatment of all chronic diseases, it improves the cardiovascular health and it may reduce the need for drugs. Exercise improves nutritional status, one of the most important determinants of survival	Exercise programs are not a part of the usual nephrology follow-up. Organization may be difficult, particularly for elderly patients	Funding the development of units for kidney care with the presence of a physiotherapist or other professional figure facilitating exercise in CKD
Personalized dialysis and nephrology care [58–62]	In all fields of medicine, personalized care is now the model of reference. Adherence is the rate limiting factor in chronic diseases and is improved when care is individualized. Incremental approaches to dialysis save resources and are planet friendly	The shortage of physicians and the increasing demands to reduce the health care budget limit the time spent with each patient, although it is a fundamental determinant of the success of care	Rethinking the field of medicine not as an industry, but as a setting for patient education and empowerment. Investing in caregiver teams

### The role of renal nurses in fostering green nephrology practices

Nurses play a crucial role in healthcare at large, including in advancing environment-friendly nephrology practices. Since nurses are involved in the preparation and accomplishment of each step of the dialysis process, this also entails a vital role in providing education on sustainable practices, both within clinical settings and in the community.

The European Dialysis and Transplant Nurses Association/European Renal Care Association (EDTNA/ERCA) has published comprehensive guidelines on how to accomplish environment-friendly nephrology practices [29]. Although particularly focused on dialysis and the day-to-day practicalities on the dialysis work floor, these also extend to a broader horizon and are not only useful for nurses but also for all others in the field, including physicians.

Nevertheless, barriers to the introduction of sustainable practices remain. Particularly for nurses, the trade-off between ergonomics and efficient therapeutic processes, and ecologically appropriate (plastic) waste handling – that can arise when, for example, considering the use of (plastic) “nurse-friendly” packaging that allows for easy handling – may create a bottleneck. It is not always easy to strike a balance between these two dimensions of kidney care, namely the efficiency with which care is delivered and the adoption of an environmentally friendly perspective in delivering care, especially under the current shortage of medical personnel worldwide and in Europe [30], which also affects kidney care [31]. Within this context, there also is a need for governments to invest more in public health and to facilitate a shift in the current kidney care model, comprising an urgent need to increase prevention, transplantation, and home dialysis, which would not only make nephrology more environment-friendly but would also address the considerable need for staff in in-centre haemodialysis.

### Increasing the uptake of organ transplantation

While home dialysis can be preferable to in-centre dialysis, kidney transplantation, out of various current KRTs, can have an even lower environmental impact; the environmental impact of kidney transplantation has the potential to be 90–95% less compared to that of HD [18, 32]. Nonetheless, kidney transplantation is an insufficiently utilised option for reducing the environmental impact of kidney disease care [17]. In most European countries, existing organ transplantation plans are not sufficiently efficient, or proved unsuccessful in meeting their targets. Governments may take an example from the success of transplantation plans in Spain [33, 34] or the UK [35, 36] and should also take the beneficial financial impact of transplantation versus dialysis into account [37]. Patient organisations can play a crucial role in creating favourable momentum to enable this transition. Such initiatives were taken in France, where patients with kidney disease engaged with multiple entities of the Government to share their suggestions and make concrete recommendations about approaches to better implement national organ transplantation plans [38, 39]. In this context, the inclusion of targets and objectives is essential, as well as engagement with the media to amplify advocacy activities. In addition, the development and implementation of a second EU Action Plan on Organ Donation and Transplantation, building on the success of the 2008–2015 action plan, would help harmonise approaches and improve the effectiveness of transplantation across EU Member States [40]. Given kidney transplantation’s low environmental impact and the dire need for a shift towards greener KRTs, improving kidney transplantation rates will not only improve patients’ quality of life, but also drastically reduce the burden of kidney care on the environment.

### Technological innovation and sustainable kidney replacement therapy

While transplantation is the preferred option for patients and the environment, not all patients will be eligible to receive a transplant, meaning that dialysis will remain the most common KRT option in the near future. Therefore, it is crucial to foster sustainable innovation in dialysis. Dialysis is characterised by notable limitations and limited technological advances since its invention in the 1940s [41]. Specifically, the dialysis filter contributes considerably to both the costs and the environmental burden linked to the current treatment of kidney failure. Indeed, the discarding of single-use dialyzers may be linked to the creation of many tonnes of medical waste [42]. Technological advancements in the production of dialysis filters, allowing them to be reused several dozens of times for the same patient, are a potential option, if safe and if cleaned filters are undamaged and usable for subsequent dialysis sessions [43], that can also reduce the environmental burden of kidney failure treatment, as fewer filters would become waste. However, to make this feasible, the alignment of EU legislation with such practices is needed. In addition, there are opportunities for reusing dialysate, now typically discarded (e.g., as wastewater), to reduce energy consumption and greenhouse gas production. Additional options exist to develop highly mobile haemodialysis systems that do not need the use of needles (e.g., by using chip-technology, dialysis filters could be drastically reduced in size, with a simultaneous increase in lifetime (several years), making them suitable for surgical implantation in patients) [44]. While such innovations are technologically achievable, significant funding for their development and exchange of international expertise are crucial if such innovations are to be translated from scientific proof-of-principle to CE-marked clinical reality. The EU should play a central role in leading sustainable innovation in kidney care, notably by earmarking funding for innovative research via EU financing mechanisms, such as the Innovative Health Initiative (IHI). It is clear that production of such devices within the EU would provide a strong export position.

### EU’s Innovative Health Initiative and the green transition in health

The EU’s Innovative Health Initiative (IHI) [45] stands as the world’s largest public–private partnership in the area of medical research, offering a perspective on how policy activities can support the transition to more sustainable healthcare practices, which also includes environment-friendly kidney care. This programme aims to deliver safe and effective health innovations across the entire care spectrum, from prevention to diagnosis and treatment, by bringing stakeholders from academia and different industries together, forestalling the prevailing tendency of working in siloes.

One of the IHI’s strategic objectives is to address the environment-friendly transition across all aspects of healthcare, even if nephrology is a very specific area within this context. Notably, the IHI call of July 2023 included two environment-related topics [46]. The first topic centred around sustainable circular development and manufacturing of healthcare products, while the second revolved around the development of new healthcare products, integrating a safe and sustainable design, with a specific focus on technologies that can minimise waste creation and enable the recycling of used devices.

### The overlooked sustainability dimensions in healthcare

While an increasing number of EU (practical) projects focus on promoting sustainable practices, including for healthcare, more can be done when it comes to EU policies like the EU Green Deal. EU policies seldom mention healthcare as an area for sustainable improvement, in contrast to other sectors. However, to facilitate this, there is a need to raise awareness of the environmental burden of healthcare amongst policy stakeholders, which can be achieved by making use of case studies. Another option is the sharing of best practices, particularly, on water conservation, the retrofitting of heat exchangers, waste disposal and the use of telemedicine and home care to limit the need for kidney patients to travel for medical consultations.

It is estimated that, by the mid-2010s, only around 10 percent of all discarded plastics had been recycled [47], and this percentage may be even lower in the healthcare sector [48]. Relying solely on recycling may be insufficient to address the entire plastic waste problem. Therefore, upstream solutions are needed to prevent waste generation. This includes evaluating the necessity of single-use plastics and eliminating unnecessary usage to reduce the need for recycling. With regard to manufacturing, product designers should collaborate with doctors and patients to understand their needs and clear demands, including for environment-friendly practices. Against this backdrop, the promoting and reinforcing of Green Public Procurement for healthcare could be a solution [49]. Green Public Procurement is a process by which public authorities seek to procure goods, services, and works with a reduced environmental impact throughout their life cycle when compared to goods, services, and works with the same primary function that would otherwise be procured. It is a pivotal strategy to encourage sustainable manufacturing and waste management within the European healthcare sector [50].

### The role of the EU in health policy

In EU policymaking circles, a tendency to focus on specific disease groups can be observed, lacking a holistic approach to health [51]. MEPs can play a role in broadening this scope by supporting a more inclusive health policy, comprising disease prevention. They can promote and raise awareness about CKD, highlighting the importance of screening and early detection in reducing the number of patients requiring dialysis. MEPs can also advocate for a new action plan to increase organ donation and transplantation through campaigns and events within the European Parliament, informing their peers and other EU institutions. Following the recent European elections in 2024, the new EU mandate presents an opportunity to focus more actively on kidney disease, with the potential for achieving significant results, similar to the successes of the European Beating Cancer Plan, which once also was a new priority for EU policymakers.

To integrate healthcare into the EU’s environmental targets (EU Green Deal, Zero Pollution Act), it is essential to demonstrate and communicate the healthcare sector's environmental impact. The kidney community should engage with environmental activist groups and MEPs involved in environmental issues to elevate this concern. There are numerous legislative opportunities within the European Parliament to address kidney disease and kidney care. Wherever appropriate, we should strive to highlight the environmental impact of these issues.

Active interest from the broader public in the EU institutions and political developments in the EU can be of value, together with lobbying activities of civil society organisations. Europeans have an important role to play in influencing the health agenda by communicating to EU policymakers the value they attach to holistic approaches to health.

## Conclusion

The 2023 European Kidney Forum illustrated the broad consensus among diverse stakeholders operating within the kidney health landscape on the critical need to adopt a more sustainable approach to kidney care in the EU. To accomplish this, the commitment of EU policymakers to improve the sustainability of the healthcare sector through EU policies, projects, and initiatives will be crucial. Moreover, the panel discussions pointed to the strong consensus between the different stakeholders in the kidney health domain on the need for action to better prevent chronic kidney disease; and to improve the EU’s organ donation and transplantation landscape for the benefit of patients, healthcare systems and the environment alike; to encourage and achieve environmentally responsible innovation in the development of KRTs; and to recognise and address the substantial environmental impact linked to the management and treatment of CKD.

## Data Availability

Data sharing not applicable to this article as no datasets were generated or analysed during the current study.
